# Post-intensive care syndrome and pulmonary fibrosis in patients surviving ARDS-pneumonia of COVID-19 and non-COVID-19 etiologies

**DOI:** 10.1038/s41598-023-32699-x

**Published:** 2023-04-21

**Authors:** Jamie L. Sturgill, Kirby P. Mayer, Anna G. Kalema, Kinjal Dave, Stephanie Mora, Alborz Kalantar, David J. Carter, Ashley A. Montgomery-Yates, Peter E. Morris

**Affiliations:** 1 Department of Microbiology, Immunology, and Molecular Genetics College of Medicine, University of Kentucky, Lexington, KY USA; 2 Department of Physical Therapy, College of Health Sciences, University of Kentucky, Lexington, KY USA; 3 Division of Pulmonary, Critical Care, and Sleep Medicine College of Medicine, Department of Internal Medicine, University of Kentucky, 740 South Limestone Street, Lexington, KY L54340536 USA; 4 College of Pharmacy, University of Kentucky, Lexington, KY USA; 5 Present Address: Kentucky Research Alliance for Lung Disease, Lexington, KY USA; 6 Division of Pulmonary, Allergy, and Critical Care Medicine, Department of Medicine, University of Alabama Birmingham, Birmingham, AL USA

**Keywords:** Health care, Medical research, Risk factors, Signs and symptoms

## Abstract

The purpose was to examine patient-centered outcomes and the occurrence of lung fibrotic changes on Chest computed tomography (CT) imaging following pneumonia-related acute respiratory distress syndrome (ARDS). We sought to investigate outpatient clinic chest CT imaging in survivors of COVID19-related ARDS and non-COVID-related ARDS, to determine group differences and explore relationships between lung fibrotic changes and functional outcomes. A retrospective practice analysis of electronic health records at an ICU Recovery Clinic in a tertiary academic medical center was performed in adult patients surviving ARDS due to COVID-19 and non-COVID etiologies. Ninety-four patients with mean age 53 ± 13 and 51% male were included (n = 64 COVID-19 and n = 30 non-COVID groups). There were no differences for age, sex, hospital length of stay, ICU length of stay, mechanical ventilation duration, or sequential organ failure assessment (SOFA) scores between the two groups. Fibrotic changes visualized on CT imaging occurred in a higher proportion of COVID-19 survivors (70%) compared to the non-COVID group (43%, p < 0.001). Across both groups, patients with fibrotic changes (n = 58) were older, had a lower BMI, longer hospital and ICU LOS, lower mean RASS scores, longer total duration of supplemental oxygen. While not statistically different, patients with fibrotic changes did have reduced respiratory function, worse performance on the six-minute walk test, and had high occurrences of anxiety, depression, emotional distress, and mild cognitive impairment regardless of initial presenting diagnosis. Patients surviving pneumonia-ARDS are at high risk of impairments in physical, emotional, and cognitive health related to Post-Intensive Care Syndrome. Of clinical importance, pulmonary fibrotic changes on chest CT occurred in a higher proportion in COVID-ARDS group; however, no functional differences were measured in spirometry or physical assessments at ICU follow-up. Whether COVID infection imparts a unique recovery is not evident from these data but suggest that long-term follow up is necessary for all survivors of ARDS.

## Introduction

One of the most severe pulmonary complications of Coronavirus Disease 2019 (COVID-19) is the development of Acute Respiratory Distress Syndrome (ARDS). The emerging data are limited by lack of succinct and collated data in patients with ARDS, but hospital censuses imply that ARDS has developed with greater frequency than with other recent adult pulmonary virus infections^1–3^. Furthermore, it is yet unclear what the actual mortality rate is from COVID pneumonia^3–6^. Patients who survive COVID-related ARDS are at risk for long term consequences of illness^7–9^. Often termed “long-COVID” these patients present with shortness of breath, brain injury (“brain fog”), sleep disorders, fevers, gastrointestinal symptoms, anxiety, and/or depression^10–12^. While still an evolving clinical scenario, the NIH has defined this syndrome as Post-Acute Sequelae of SARS-CoV-2 infection (PASC)^13^.

For COVID-19 pneumonia survivors who required mechanical ventilation, it is yet unclear how long functional limitations will remain. One of the most significant manifestations of PASC is the shortness of breath, which may be multifactorial but includes pulmonary fibrotic changes as a potential etiology. In addition, patients surviving ARDS from all-causes are at risk of suffering impairments in physical, emotional, and cognitive domains of health, referred to as Post Intensive Care Syndrome (PICS)^14,15^. Early reports in patients that have survived severe COVID-19 demonstrate a potential for high occurrence of symptoms and impairments related to PICS^16^.

Ground glass opacities (GGOs), which is a radiographic finding indicative of abnormal lung parenchyma, is frequently associated with COVID pneumonia and resultant ARDS. This finding may represent interstitial inflammation and partial alveolar filling^17^. This radiologic presentation is consistent with the underlying pathophysiology of ARDS which involves extensive pulmonary inflammation and exudative alveolar filling^18,19^. While GGOs are frequently seen on imaging of most ARDS patients^20^, the estimation of the exact incidence in COVID19 pneumonia-related ARDS is limited by the low numbers of patients included in the medical literature to date. Furthermore, the length of time in which these radiographic changes persist after the acute phase of ARDS is an important, yet understudied clinical question. The limitations of current literature prompted this investigation into assessing the incidence of Chest CT scan-related pulmonary fibrotic changes in COVID-19 ARDS survivors and ARDS survivors due to other etiologies. To that end, a retrospective cohort study was conducted in patients who visited our ICU recovery clinic.

## Methods

### Study design

The study is a retrospective analysis of the medical records of adult patients surviving acute respiratory distress syndrome (ARDS) and receiving follow-up care in the ICU Recovery Clinic at the University of Kentucky^21^. The study was approved by the institutional review board at the University of Kentucky (Medical Expedited Review #47751) with informed consent waived due to the study design. The study complies with relevant ethical regulations and performed under the Declaration of Helsinki—ethical principles for medical research involving human subjects.

Two groups were included in the study: (1) patients with severe to critical COVID-19 disease as defined by the World Health Organization severity scale and laboratory confirmed SARS-CoV-2 infection by qPCR (COVID-19) and (2) patients with acute respiratory failure developing ARDS due to an acute pulmonary or extrapulmonary insult (Non-COVID). Adult patients (≥ 18 years of age but less than 99 years of age) were eligible for the study if they had required invasive mechanical ventilation for at least 72 h and received a Computed Tomography (CT) scan of the chest between 1 and 3 months after hospital discharge. Patients were excluded from the analysis if they were a prisoner, pregnant, had a chronic tracheostomy or were receiving medication to treat pulmonary fibrosis prior to the hospital admission for ARDS. Patients in the non-COVID group followed up in the ICU Recovery Clinic and had a CT chest performed from January 28, 2016 to January 12, 2022, while patients in the COVID-19 group follow-up occurred from June 1, 2020 to March 1, 2022. The two groups were extracted from the ICU Recovery Clinic database.

### Dependent variables

The primary dependent outcome was a visualization of pulmonary fibrotic changes on the post-hospital CT imaging, quantified as a binomial variable (yes, no). The same approach was utilized for ground-glass opacities (GGO). Due to heterogeneity in assessment of CT imaging in clinical practice, images were assessed with two approaches: (1) radiologist report from clinical practice and (2) re-examined by two blinded Intensivists with experience in ICU Recovery (AGK-board certified in Critical Care; and KD-board certified in Critical Care and Pulmonary). The radiology reports and images were extracted from the EHR by a research team member and coded to maintaining blinding of Intensivists. The Intensivists scored four questions on a standardized scoring document for binary outcomes (yes, no) in the following order: (1) GGO present on the imaging; (2) fibrotic changes on imaging; (3) GGO language reported on the radiologist report; and (4) fibrotic changes on the radiologist report. Fibrotic changes on the radiologist reported included terms “pulmonary fibrosis”, “scarring”, “bronchiectasis”, and “honeycombing”. Intensivists independently reviewed images prior to examining the report to prevent interpretation biases. Each Intensivist examined 75% of the sample such that 50% overlapped to assess agreement between raters.

Secondary patient outcomes were measured on the same day as the CT scan (standard of care for ICU Recovery Clinic) including forced vital capacity (FVC) and forced expiratory volume in one second (FEV_1_) measured as absolute and percentage of predicted on spirometry. In addition, patients participated in the Core Outcome Measurement Set developed for patients surviving acute respiratory failure^22^ and recommended by National Heart Lung and Blood Institute’s working group^23^: cognitive function measured by Montreal Cognitive Assessment (MoCA, ≤ 23 denoting mild cognitive impairment [MCI]), health related quality of life (HrQOL) on a self-report visual analog scale (VAS) from the EuroQol-5D instrument which ranks from 0 as the worst health imaginable to 100 as the best health imaginable, and emotional health reported on self-report questionnaires including Impact of Events Scale-revised (IES-R) and Hospital Anxiety and Depression Scale (HADS). The IES-R is a 22-item measure (0–88) assessing emotional distress caused by traumatic events with score > 33 representing a provisional diagnosis of post-traumatic stress disorder (PTSD). HADS is a 14-item self-report measure (0–42) for post-hospital emotional health with cut-off scores of > 8 for each category representing anxiety or depression. Clinicians in the ICU Recovery Clinic may also administer the 6-min walk test (6MWT). Secondary outcome measures performed 1–3 months after hospital discharge in the ICU Recovery Clinic as part of routine care were considered for analyses. A composite outcome of 90-day hospital readmission and/or emergency department utilization was extracted from electronic health record (EHR).

### Demographics and past medical history (PMH) variables

Independent variables were extracted from the EHR included age, sex, body mass-index (BMI), race/ethnicity, tobacco use status, Charlson Comorbidity Index (CCI), and a past medical history of chronic lung disease, asthma, and/or obstructive sleep apnea.

### Critical Illness and hospital variables

ICU and hospital variables were extracted from the EHR: mechanical ventilation (yes or no), high-flow nasal cannula (yes or no), mechanical ventilation (MV) duration (days), duration of receiving oxygenation of HFNC and/or MV, and the highest fraction of inspired air (FiO2), highest positive end-expiratory pressure (PEEP), and driving pressure measured in the first 72 h of ventilation to represent initial ICU treatment approach. Severity of illness was quantified with sequential organ failure assessment (SOFA) as worse score in first 48 h of ICU admission as a surrogate marker of initial acuity. Binomial variables (yes, no) were extracted for receipt of continuous renal replacement therapies (CRRT), extracorporeal membrane oxygenation (ECMO), steroid administration, vasopressor or inotrope, or a continuous administration of a neuromuscular blocker. Sedative status measured by Richmond Agitation Sedation Scale (RASS) as a mean in the first 72 h of ICU admission as a surrogate marker of sedative status. Investigational therapies were assessed for patients in the COVID group including the receipt (yes, no) of Hydroxychloroquine, Tocilizumab, Baricitinib, Remdesivir, and/or convalescent plasma.

### Statistical analysis

Descriptive statistics and data visualization were performed to assess the central tendencies (mean or median) and variations (standard deviation [SD] or interquartile range [IQR]). Normality was assessed by the Shapiro–Wilk test and the appropriate parametric and non-parametric statistics were performed. The absolute agreement between the two Intensivists rating of the primary dependent outcomes were assessed with inter-rater reliability testing (intra-class correlation coefficient [ICC]). Then, the agreement between the radiologist report and the Intensivists assessment for the primary outcomes were assessed with absolute agreement via inter-rater reliability testing (ICC). Disagreements on the primary outcome between radiologist report and intensivist were discussed and consensus achieved (AK, KD, AMY, PEM) to confirm primary dependent outcomes. Independent t-tests (Welch’s correction for unequal variance) and Chi-square tests were performed to determine difference between the COVID and non-COVID groups for independent and dependent variables. Chi square tests were performed to examine the primary null hypothesis: occurrence of fibrotic changes is not different between the two groups. Secondarily, independent t-tests (Welch’s correction for unequal variance) and Chi-square tests were performed to determine difference between independent and dependent variables for patients with fibrotic changes on CT regardless of diagnosis group compared to patients without fibrosis. Statistics were performed using SPPS version 27 (IBM, Armonk, NY, USA) GraphPad Prism (GraphPad, San Diego, CA, USA) with significant set at p < 0.05.

### Ethics approval and consent to participate

The study was approved by the institutional review board at the University of Kentucky with informed consent waived due to the study design (MEDXP # 47751).

## Results

### Descriptive

Ninety-four patients with mean age of 53.2 ± 13.2 and 51% male were included in the study (Table 1). Sixty-four patients met inclusion for COVID-19 group and 30 were eligible in the non-COVID group. There were no statistical differences in age, sex, SOFA scores, or comorbidity history between the two groups (Table 1). However, the total duration of supplemental oxygen, BMI, peak FiO2, PEEP, and driving pressure were statistically different between the two groups (Table 1). Patients in the COVID-19 group were more likely to receive steroids.

**Table 1:** Demographic and clinical data for patients surviving ARDS.

Parameter	COVID-19 n = 64	Non-COVID n = 30	T-test or Chi-square, p = 0.05
Age, years, mean ± SD	54 ± 13	50 ± 13	p = 0.14
Male, n (%)	32 (50)	16 (53)	p = 0.77
Race/ethnicity			
Caucasian/White, n (%)	48 (75)	30 (100)	p = 0.002^A^
African America/Black, n (%)	9 (14)	0 (0)	
Other, n (%)	7 (11)	0 (0)	
Tobacco use			
Current, n (%)	10 (16)	13 (43)	p = 0.38^B^
Former, n (%)	19 (30)	5 (17)	
Never, n (%)	33 (52)	12 (40)	
Body mass index (kg/m^2^), mean ± SD	35.9 ± 8.4	32.6 ± 8.3	p = 0.046
Charlson comorbidity index	2.7 ± 1.9	2.6 ± 2.1	p = 0.85
Chronic lung disease, n (%)	10(16)	11 (37)	p = 0.033
SOFA, mean ± SD	11.3 ± 2.5	10.7 ± 2.6	p = 0.34
MV, yes, %	64 (100)	30 (100)	
MV duration, mean ± SD	17.3 ± 12.4	15.1 ± 11.2	p = 0.33
Oxygenation (HFNC + MV) days, mean ± SD	20.8 ± 12.6	16.2 ± 11.4	p = 0.048
FiO2 (peak 72 h), mean ± SD	90.9 ± 14.3	78.8 ± 22.5	p = 0.009
PEEP (peek 72 h), mean ± SD	13.6 ± 3.2	11.5 ± 4.4	p = 0.030
Tracheostomy, n (%)	24 (38)	14 (47)	p = 0.40
ECMO, n (%)	9 (14)	4 (13)	p = 0.92
CRRT, n (%)	9 (14)	4 (13)	p = 0.92
Steroids, n (%)	58 (91)	16 (53)	p < 0.001
Vasopressor/inotropes, n (%)	45 (70)	19 (63)	p = 0.50
NMB, n (%)	24(38)	9 (30)	p = 0.48
RASS (mean 72 h), mean ± SD	− 3.4 ± 1.2	− 2.9 ± 1.5	p = 0.16
COVID-19 therapies			
Hydroxychloroquine	5 (8)		
Tocilizumab	14 (22)		
Remdesivir	38 (59)		
Convalescent plasma	11 (17)		
ICU LOS	23.2 ± 14.8	20.9 ± 13.9	p = 0.394
Hospital LOS	33.8 ± 17.6	26.9 ± 15.5	p = 0.09
Discharge destination			
LTAC	5 (8)	9 (30)	
Acute	36 (56)	6 (20)	p = 0.26^C^
Home with HH	12 (19)	6 (20)	
Home	11 (17)	9 (30)	

*SOFA* sequential organ failure assessment, *MV* mechanical ventilation, *FiO2* fraction of inspired oxygen, *PEEP* positive end expiratory pressure, *ECMO* extracorporeal membrane oxygenation, *AKI* acute kidney injury, *CRRT* continuous renal replacement therapy, *NMB* neuromuscular blocker, *RASS* Richmond Agitation Sedation Scale.

^A^Fisher’s exact test assessing Caucasian/White vs combined African American/Black and Other.

^B^Fisher’s exact test assessing combined Current and Former vs Never tobacco use.

^C^Fisher’s exact test comparing discharge to Home + Home with HH compared to discharge to facility (acute + LTAC).

### Reliability and agreement of CT imaging interpretations

Strong agreements were observed between the two Intensivist for interpretations of GGO with only two disagreements on 50 images (96%, ICC = 0.94 [95% CI 0.89–0.97]) and for fibrotic changes with one disagreement (98%, ICC = 0.97 [95% CI 0.96–0.99]). There were 15 disagreements between the radiologist and Intensivists on 94 images for presents of GGO (84%, ICC = 0.67 [95% CI 0.52–0.76]), and six disagreements were recorded for fibrotic changes on CT images (94%, ICC = 0.86 [95% CI 0.79–0.90]). Consensus was achieved suggesting that radiology reports omitted terms to describe present of fibrotic changes on 6 reports.

### Primary outcomes

Patients surviving ARDS due to COVID-19 were more likely to have fibrotic changes on CT images compared to patients surviving non-COVID-related ARDS (Table 2). In addition, patients in the COVID group had a higher occurrence of GGO on imaging. Statistically, there was a shorter time to CT scan in COVID-19 (89 ± 38 days) group compared to non-COVID group (117 ± 55 days). Patients with fibrotic changes regardless of diagnosis group were older, more likely to be male, had a lower BMI, had longer time requiring oxygenation (MV + HFNC duration), and had worse sedative status (Table 3).

**Table 2:** Primary and secondary outcomes grouped by etiology of ARDS.

Parameter	COVID-19 n = 64	Non-COVID n = 30	T-test or Chi-square p = 0.05
90-day negative composite	15 (23)	7 (23)	p = 0.99
CT findings			
Fibrotic changes, n (%)	45 (70)	13 (43)	p = 0.022
Ground-glass opacity, n (%)	52 (81)	12 (40)	p < 0.001
Time to CT scan, days	89 ± 38	117 ± 55	p = 0.023
Spirometry	n = 55	n = 24	
FEV1	2.2 ± 0.7	2.1 ± 0.7	p = 0.84
FEV1% predicted	67 ± 17	66 ± 20	p = 0.83
FVC	2.6 ± 0.9	2.9 ± 0.9	p = 0.081
FVC % predicted	63.9 ± 19	71 ± 20	p = 0.157
6 MWT (n)	n = 51	n = 8	
Distance, meters, mean ± SD	229 ± 131	260 ± 169	p = 0.63
6 MWD %	44 ± 26	46 ± 32	p = 0.87
Cognitive and emotional outcomes	n = 52	n = 25	
MOCA, mean ± SD	25.6 ± 3.4	24.0 ± 3.6	t = 1.92, p = 0.062
MCI (cutoff < 25) yes, n (%)	19 (37)	12 (54)	p = 0.151
EQ-5D VAS, mean ± SD	69.3 ± 16	55.3 ± 20	t = 2.94, p = 0.006
IES-R, mean ± SD	26.1 ± 22	36.8 ± 24	t = 1.88, p = 0.066
IES-R cutoff > 33, yes, n (%)	17 (32)	15 (60)	p = 0.047
HADS-anxiety, mean ± SD	6.5 ± 4.8	9.3 ± 5.5	t = 2.18, p = 0.035
HADS-anxiety cutoff > 8, yes, n (%)	15 (29)	16 (64)	p = 0.012
HADS-depression, mean ± SD	5.9 ± 4.1	8.1 ± 5.5	t = 1.78, p = 0.083
HADS-depression cutoff > 8, yes, n (%)	13 (25)	12 (48)	p = 0.069

*ICU* intensive care unit, *LOS* length of stay, *LTAC* long-term acute care facility, *Acute* acute rehabilitation facility, *Home with HH* home with home health services, *ER* emergency room visit, *CT* commuted tomography, *FVC* forced vital capacity (FVC), *FEV1* forced expiratory volume in one second, *MOCA* Montreal Cognitive Assessment; *MCI* mild cognitive impairment, *EQ-5D VAS* Euro-Quality of Life5 Dimension-Visual Analog Scale, *IES-R* impact of events scale revised, *HADS* hospital anxiety and depression scale.

**Table 3:** Clinical variables and patient-outcomes grouped by fibrotic changes on CT images at short-term follow-up.

Parameter	Fibrotic changes n = 58	No fibrotic changes n = 36	T-test/Chi-square p = 0.05
Age, years, mean ± SD	55.5 ± 13	49.4 ± 12.4	p =0.018
Male, n (%)	37 (64)	11 (31)	p = 0.003
BMI, kg/m^2^, mean ± SD	33 ± 7	37.6 ± 9.5	p = 0.035
Charlson comorbidity index, mean ± SD	2.8 ± 1.9	2.3 ± 2.1	p = 0.135
MV duration, days, mean ± SD	17.6 ± 10.5	15.0 ± 14	p = 0.067
Fi0_2_, highest in first 72 h, mean ± SD	87 ± 17	87 ± 20	p = 0.965
PEEP, highest in first 72 h, mean ± SD	12.6 ± 3.2	13.5 ± 4.6	p = 0.315
Oxygenation (HFNC + MV, days, mean ± SD	21 ± 11	16 ± 14	p = 0.003
SOFA, mean ± SD	11.5 ± 2.5	10.6 ± 2.4	p = 0.089
RASS in first 72 h, mean ± SD	− 3.5 ± 1.2	− 2.8 ± 1.3	p = 0.005
ICU LOS, mean ± SD	24.4 ± 14	19.3 ± 15	p = 0.015
Hospital LOS, mean ± SD	34 ± 16	27 ± 18	p = 0.016
90-day readmit/ER, yes, n (%)	12 (21)	10 (28)	p = 0.460
Spirometry (n)	52	27	
FEV1	2.2 ± 0.7	2.1 ± 0.7	t = 0.2, p = 0.84
FEV1% predicted	67 ± 17	67 ± 19	t = 0.2, p = 0.86
FVC	2.7 ± 0.9	2.7 ± 0.9	t = 0.5, p = 0.61
FVC % predicted	64 ± 19	70 ± 20	t = 1.5, p = 0.13
6MWT (n)	39	20	
Distance, meters, mean ± SD	228.6 ± 121	243 ± 162	t = 0.10, p = 0.92
% of predicted, % mean ± SD	44 ± 24	46 ± 31	t = 0.09, p = 0.92
QOL and cognitive outcomes (n)	n = 48	n = 28	
MOCA, mean ± SD	25 ± 3.3	25 ± 4.0	t = 0.54, p = 0.589
MCI (cutoff < 25) yes/n (%)	18 (38)	13 (46)	p = 0.468
EQ-5D VAS, mean ± SD	67 ± 19	62 ± 17	t = 1.08, p = 0.281
Emotional outcomes (n)	n = 48	n = 28	
IES-R, mean ± SD	26 ± 22	36 ± 25	t = 1.688, p = 0.098
IES-R cutoff > 33, yes, n (%)	15 (31)	17 (61)	p = 0.016
HADS-anxiety, mean ± SD	6.9 ± 5.2	8.3 ± 5.0	t = 1.10, p = 0.275
HADS-anxiety cutoff > 8, yes, n (%)	18 (38)	13 (46)	p = 0.477
HADS-depression, mean ± SD	6.0 ± 4.5	7.7 ± 4.9	t = 1.52, p = 0.135
HADS-depression cutoff > 8, yes, n (%)	14 (29)	11 (39)	p = 0.450

*MOCA* Montreal Cognitive Assessment, *MCI* mild cognitive impairment, *VAS* visual analog scale, *HADS* hospital anxiety and depression scale, *IES-R* impact of events scale-revised, *PTSD* post-traumatic stress disorder.

### Secondary outcomes

There were no differences in FEV1, FEV1%, FVC, or FVC% between the COVID and non-COVID groups (Table 2). Patients from the cohort achieved a mean of 233 ± 139 m on the 6MWD which equates to an average of 44 ± 27% on predicted distances but performance was not different between the COVID-19 and non-COVID-19 survivors (Table 2). Performance on spirometry and 6 MWT were also not different based on the presence of fibrosis regardless of the group (Table 3).

Patients in COVID-19 group self-reported better quality of life compared to the non-COVID group had (69 vs 55, p = 0.006, Table 2). Occurrence of MCI in the entire cohort was 40% (n = 31/77) and statistically not different between groups (Table 2). The occurrence of anxiety (40%, n = 31/77), depression (32%, n = 25/77) and PTSD (42%, n = 32/77) were high for the entire cohort; patients in the non-COVID group had statistically higher occurrences of anxiety and PTSD (Table 2). Twenty-two patients (29%) had at least one negative-composite outcome within 90-days of hospital discharge which was not different for COVID (23%) and non-COVID (23%, p = 0.99). When grouping the cohort according to pulmonary fibrosis, there were no differences in quality of life, MCI, anxiety, depression, or negative composite outcome (Table 3). Patients with fibrotic changes on CT imaging did have lower occurrence of provisional diagnosis of PTSD on IES-R (30% vs 61%, Fisher’s exact, p = 0.016).

## Discussion

Survivors of critical illness due to COVID-19 with subsequent ARDS have been reported to demonstrate Chest CT findings consistent with pulmonary fibrosis measured 3-months after hospital discharge^24–27^. The findings from this single-center study confirm the high occurrence of fibrotic changes on Chest CT for COVID-19 pneumonia with ARDS, which occurred at a higher frequency when compared to a group of patients with non-COVID ARDS etiologies. The results should be interpreted with caution due to the inherent biases including study design, survivorship biases, and inability to match the sample sizes of the two cohorts. The two cohorts had a similar severity of illness with nearly identical SOFA scores and ICU length of stays with all 94 patients receiving care in an ICU Recovery Clinic, and thus supports comparative analyses that may enhance clinical practice. The physiologic nature of COVID-19 as well as the approach to ventilation and pharmaceutical treatment of ARDS for COVID-19 may place these survivors at a higher risk of pulmonary fibrosis.

The two groups of ARDS patients demonstrated a similar degree of physical, emotional, and cognitive impairments related to PASC and PICS, which begs the question that PASC may be a form of PICS itself. Distances achieved on 6 MWT performed approximately 2 months after hospital discharge were significantly lower than the predicted distances^28^ and were similar to historical reports for non-COVID ARDS survivors at same time-point (320 ± 138^29^; 281 [55–454]^30,31^). These data indicate that despite the underlying cause, all patients surviving ARDS could conceivably benefit from a post-ICU follow up to assess and address long term sequela of critical illness.

Previous reports demonstrated that COVID-related ARDS frequently describe morphological and functional changes of the lungs; however, there have been limited comparisons of the incidence of post-COVID19 ARDS-related pulmonary fibrosis to the development of pulmonary fibrosis in survivors of other ARDS etiologies^32^. One study found that 94.5% of COVID-related ARDS had GGOs compared to only 45.3% of ARDS secondary to H1N1^32^. In a study of COVID-19 patients who did not require ICU admission approximately half demonstrated impairments in DLCO. A recent report revealed that 72% patients with severe COVID-19, who required mechanical ventilation, developed fibrotic lung changes on Chest CT, which was significantly higher than COVID-19 patients not requiring MV^24^. Findings in our study are consistent with the previously reported rate of occurrence of pulmonary fibrosis in post-COVID ARDS, but the previous reports often do not include a non-COVID ARDS comparison group. In McGroder’s report, the 6MWD did associate with dyspnea scores but not with radiographic abnormalities^24^. In our study, pulmonary function and physical performance were impaired in both groups regardless of presence of fibrosis and regardless of diagnosis.

We could not control for missing data and it should be noted that there is potential for follow-up biases e.g. patients attending follow-up could be hypothetically less sick since they were are able to travel back to clinic and potentially patients have better social support to come to clinic. These preliminary findings, however, do provide evidence that some survivors of ARDS require detailed follow-up to assess symptoms. Frequent demonstration of impairments suggests that the promotion of a treatment plan to enhance recovery and mitigate symptoms related to Post-Intensive Care Syndrome, might be of benefit for similar survivors. Findings from our study confirm that survivors of both ARDS groups have cognitive impairment, anxiety, depression, and PTSD which is consistent with previously reported data in patients surviving critical illness^33^. However, patients in the non-COVID group reported lower self-report scores on quality of life and had higher occurrence of anxiety compared to the COVID-19 cohort. At this juncture, it is unclear how much of a role the heightened COVID media attention affects symptom description whether it alleviates or accentuates symptom severity and quality of life reporting. We may speculate from clinic that patients surviving COVID-19 may have received better clinic and social support including specific attention to discharge education.

Interestingly, patients with fibrotic changes regardless of diagnosis had lower BMI. We were unable to capture patient-self-inflicted lung injury parameters (P-SILI) or ventilator dyssynchrony in our EHR, but sedation and paralytics administration at our institution could contribute to P-SILI. Finally, twenty-one percent of the current study’s population had medical histories reporting a chronic lung disease. Consistent with other post-COVID radiographic reports and other post ARDS reports, the role of a pre-hospitalization existence of a chronic lung disease has not been fully weighed. These patients often receive ARDS-ICU care far from home in the US, and therefore, access to pre-hospital pulmonary function testing or imaging may not be feasible. Whether the presence of underlying, pre-existing chronic lung disease is an independent factor to the post-COVID pneumonia development of fibrosis is still unclear and warrants further study.

Whether or not all of these radiographic lesions resolve over time or become permanent or progress is currently not clear from these data nor from data in the medical literature. The limitations of this study include the retrospective design of the study that precludes associations indicative of casualty. Furthermore, the design introduces the potential for selection and misrepresentation biases. These data were based on a cohort of patients who were seen in the ICU recovery clinic at a tertiary academic medical center and thus, are not drawn from all ICU survivors. Based on our previous findings, we know that patients who reside in rural areas and who required an extended ICU LOS, are less likely to attend post-ICU clinic, thus introducing a selection bias in the data^21^. The study examined only survivors of critical illness, therefore the results present the potential for survivorship bias. The approach to assess pulmonary fibrosis as a binomial variable, may undervalue the spectrum of fibrosis severity. As well, there is potential for differences in how different Radiologists may interpret CT images as being consistent with a pulmonary fibrotic change. Finally, due to the sample size and the inherent biases aforementioned, we did not perform a multivariate logistic regression.

Overall limitations for this area of post-hospital evaluation and care of ARDS survivors is currently somewhat limited in the ability to predict accurately the incidence of post-ARDS fibrosis and the impact of post-ARDS fibrosis on functionality and long-term survivorship. Future prospective investigations could improve this area of medical care by incorporating several parameters into the approach in ICU Survivors clinics. These parameters would be to capture the pre-hospitalization existence of lung disease. Since the future task will be to relate the degree of fibrotic changes on Chest CT scans of ARDS survivors to their functional output, more organization of the timing of outpatient CT scans would benefit the cross-study comparisons of the rate of resolution of several Interstitial Lung abnormalities. To optimize the characterization of fibrotic changes lung parenchymal changes, the field will need to strongly recommend that post-ARDS imaging not only be organized in terms of timing post hospital discharge but to also unify the test ordered by selecting the high-resolution modality for the requested Chest CT. As well, attempts by ICU Recovery Clinic staff should be made to assess the survivors’ degree of pre-hospitalization functionality. The WHO-DAS may be a potential tool to establish a uniform approach to assessing pre-hospitalization function in ICU survivors^34,35^.

Several aspects of patient symptomatology may also require specific attention such as quantification of cough and whether readmission to hospital for dyspnea has occurred since last visit or initial hospitalization. For optimization of patient selection for potential antifibrotic therapies, early characterization of survivors into a recognized trajectory pattern will be meaningful to assess various approaches to restore functionality. Overall, with all potential planned interventions, a close recording of participation or lack of participation in pulmonary rehabilitation efforts will be an important parameter to strictly record. Such information will aid the interpretation of any functional improvements and help correlate a functional change to either the resolution or persistence of chest CT interstitial lung abnormalities.

Our study is not without limitations. Primarily, the retrospective study design limits the generalizability due to potential for misrepresentation, selection, and survivorship biases. Thus, the findings are not causative and should be interpreted with caution. Moreover, the approach to the analysis of the CT introduces a few limitations. First, there is potential significant heterogeneity in the approach to assessing changes on CT scans by different radiologists. Second, the CT scans were utilized from clinical practice and not based on quantifying the severity of fibrotic changes. Thus, subjectivity and heterogeneity by multiple radiologist reduce the rigor of the study and introducing variability and confounding the results. We, however, elected to focus on the clinical CT reports as evidence from clinical practice. This further highlights the need to standardize post-ARDS radiologic and physical examination techniques. Finally, the study is limited by small sample sizes in the two groups that included non-concurrent study times.

## Conclusions

Patients surviving ARDS regardless of underlying cause have symptoms and impairments related to PICS. In addition, we demonstrate that patients surviving COVID-19 pneumonia have an increased evidence of fibrotic changes compared to non-COVID pneumonia ARDS patients however the long-term pulmonary physiologic consequences remain to be seen. Considerable future efforts to enable the capture of post-COVID ARDS survivors’ symptoms, imaging, pulmonary function and functional assessments may ultimately allow for design optimization of interventional studies to promote return of function.

## Data Availability

The minimum data set are presented in the manuscript. Data are available upon reasonable request at the discretion of the corresponding author.
